# Outer Membrane Protein A (OmpA) of *Shigella flexneri* 2a, Induces Protective Immune Response in a Mouse Model

**DOI:** 10.1371/journal.pone.0022663

**Published:** 2011-07-26

**Authors:** Debasis Pore, Nibedita Mahata, Amit Pal, Manoj K. Chakrabarti

**Affiliations:** Division of Pathophysiology, National Institute of Cholera and Enteric Diseases, Beliaghata, Kolkata, West Bengal, India; Indian Institute of Science, India

## Abstract

**Background:**

In our earlier studies 34 kDa outer membrane protein (OMP) of *Shigella flexneri* 2a has been identified as an efficient immunostimulant.

**Key Results:**

In the present study MALDI-TOF MS analysis of the purified 34 kDa OMP of *Shigella flexneri* 2a shows considerable sequence homology (Identity 65%) with the OmpA of *S. flexneri* 2a. By using the specific primers, the gene of interest has been amplified from *S. flexneri* 2a (N.Y-962/92) genomic DNA, cloned in pET100/D-TOPO® vector and expressed using induction with isopropyl thiogalactoside (IPTG) for the first time. Immunogenicity and protective efficacy of the recombinant OmpA has been evaluated in an intranasally immunized murine pulmonary model. The recombinant protein induces significantly enhanced protein specific IgG and IgA Abs in both mucosal and systemic compartments and IgA secreting cells in the systemic compartment (spleen). The mice immunized with OmpA have been protected completely from systemic challenge with a lethal dose of virulent *S. flexneri* 2a. Immunization with the protein causes mild polymorphonuclear neutrophil infiltration in the lung, without inducing the release of large amounts of proinflammatory cytokines.

**Conclusion:**

These results suggest that the OmpA of *S. flexneri* 2a can be an efficacious mucosal immunogen inducing protective immune responses. Our findings also demonstrate that antibodies and Th1 immune response may be associated with the marked protective efficacy of immunized mice after intranasal shigellae infection.

## Introduction

Shigellosis, an important etiological agent of bacillary dysentery in humans is caused by *Shigella*, a Gram-negative bacterium which belongs to the family *Enterobacteriaceae* [1]. Each year millions of cases occur globally, with the majority occurring in children in developing countries, and over 600,000 cases resulting in death [2]. Antibiotics are generally effective against shigellosis, however, the increasing level of antibiotics resistance found in *Shigella* isolates, even to the newest antibiotics [3] and oral rehydration therapy alone is not adequate in treating shigellosis has made it necessary to develop alternate treatment and prevention strategies. Therefore, the World Health Organization has given high priority to the development of a safe and effective vaccine against shigellosis [1].

Many approaches for the development of *Shigella* vaccines have been attempted [4]–[10]. Unfortunately, no practical vaccine is available so far. The current vaccine candidates are either not sufficiently attenuated or immunogenic enough [5], [11], demonstrating the identification of further attenuation or novel protective antigen is indispensable. Recently, immunoproteome analysis of *S. flexneri* has shown that more protective antigens, which can be screened from immunogenic outer membrane protein, may be selected as vaccine candidates [12]–[14].

Towards vaccine approach, previously from our laboratory among different outer membrane proteins (OMPs) of *Shigella flexneri* 2a [15], the gel cut 34 kDa OMP has been identified as the major protective antigen [16]. Recently the 34 kDa OMP of *S. flexneri* 2a has been purified and characterized. It has been found that the protein is crossreactive and antigenically conserved among *Shigella* spp., the epitope is surface exposed on the intact bacterium [17]. Upon further characterization, the protein has been shown to stimulate macrophages through a TLR2-dependent mechanism [18]. Moreover, 34 kDa protein has been found to up regulate the expression of adaptor protein MyD88, p38 MAP kinase, NF-κB, production of type-1 cytokines and chemokines as well as other molecules (MHC II, CD40 and CD80) known to modulate the adaptive response towards Th1 type in macrophages [18], thus linking the innate and adaptive responses to the antigen. All these features of the 34 kDa OMP demonstrate that this protein could successfully be used as suitable candidate for vaccine development against shigellosis.

In our previous study, purification of the *S. flexneri* 2a 34 kDa OMP to apparent homogeneity has been achieved by molecular-sieve and ion exchange chromatographic techniques with a yield of 100 µg per litter culture. As the yield is very low and protective efficacy of the purified protein has not been tested in an animal model, therefore in continuation to our previous finding [17] the present study has been undertaken to clone and overexpress the 34 kDa OMP of *S. flexneri* 2a. For this purpose MALDI-TOF MS analysis of the purified 34 kDa OMP has been performed, which identifies the protein as OmpA of *S. flexneri* 2a. Based on the corresponding *ompA* gene sequence of *S. flexneri* 2a, oligonucleotide primers have been designed. The gene encoding the OmpA has been amplified by PCR, cloned in pET100/D-TOPO® vector, sequenced and expressed in BL21 Star™(DE3) using induction with isopropyl thiogalactoside. The present communication also deals with the evaluation of immunogenicity and protective efficacy of the recombinant OmpA in mice pulmonary pneumonia model. To understand the molecular immunological mechanism, local and systemic antibody responses to the protein in serum and mucosal compartment have been studied. Moreover, histology of mice lung tissues and cytokine responses (macrophage inflammatory protein-2; MIP-2, interleukin six; IL-6, interferon gamma; IFN-γ, tumor necrosis factor alpha; TNF-α) in the lung lavage fluid have been compared among control and immunized mice. Our results indicate that nasal immunization with the recombinant OmpA is an effective vaccine approach to induce protective immune responses in mice against infection by virulent *S. flexneri* 2a.

## Materials and Methods

### Bacterial strains and culture

*Shigella flexneri* 2a (N.Y-962/92) was obtained from the Pathophysiology Division of National Institute of Cholera and Enteric Diseases, Kolkata, India.

### Animal

BALB/c mice, originally obtained from Jackson Laboratories (Bar Harbor, ME), were bred and reared in the animal facility at the National Institute of Cholera and Enteric Diseases, Kolkata, India. The experiments with animals were conducted in accordance with the Animal Ethical Committee guidelines of National Institute of Cholera and Enteric Diseases, Kolkata, India (approval no. 18/7-6-2000).

### Purification of 34 kDa OMP

34 kDa OMP was purified to apparent homogeneity from *S. flexneri* 2a as described previously [17].

### MALDI-TOF MS sequencing of the purified 34 kDa OMP

MALDI-TOF MS of purified 34 kDa OMP was performed to evaluate the amino acid sequence of the full-length protein. For this, the purified protein was reduced and alkylated with iodoacetamide, i.e. carbamidomethylated, and digested with trypsin that cleaves after lysine and arginine residues. The resulting peptides were concentrated on a ZipTip micropurification column and eluted onto an anchorchip target for analysis on a Bruker Autoflex III MALDI TOF/TOF instrument at Alphalyse, (Denmark). The peptide mixture was analyzed in positive reflector mode for accurate peptide mass determination and 5–10 of the peptides selected for analysis by MS/MS fragmentation for partial peptide sequencing. The MS and MS/MS spectra were combined and used for a database search in an in-house protein database using the Mascot software.

### Liposome swelling assay of 34 kDa OMP

Liposome swelling assay was carried out essentially as described by Nikaido *et al.*, 1991 [19], by using a mixture of acetone washed egg phosphatidylcholine and dicetylphosphate as the phospholipid with the 34 kDa protein. Portions (13 µl) of these liposomes and 0.6 ml of iso-osmotic L-arabinose in 5 mM Tris-HCl (pH 7.4) were mixed rapidly in a cuvette, and the A_400_ was recorded.

### Isolation of genomic DNA

Genomic DNA of *S. flexneri* 2a was prepared using the method described by Sharma and Sing [20].

### Cloning and expression of the OmpA

From the GenBank, the sequence coding for the OmpA (Accession No. NP_836665) of *S. flexneri* 2a was retrieved. To amplify the gene, the following primers were designed: F 5′-CACCATGAAAAAGACAGCTATC- 3′ and R 5′- TTAAGCCTGCGGCTGAGTTA-3′. PCR amplified product was resolved in 1% agarose gel by electrophoresis and analyzed using Gel-Doc (Bio-Rad). The PCR amplified product of *ompA* gene was ligated to commercial pET100/D-TOPO® linearized vector (Invitrogen). Ligated product was transformed into One Shot® TOP10 chemically competent *E. coli* cells by heat shock. The recombinant transformants were selected using ampicillin (100 µg/ml) on LB agar plates. The colonies containing the recombinant plasmid was identified and confirmed by DNA sequencing. The plasmid from the correct transformant was isolated from an overnight culture of recombinant *E. coli* Top 10 cell by using alkaline lysis protocol [21] and then transformed into BL21 Star™ (DE3) One Shot® Chemically Competent *E. coli* cells by heat shock. The transformed *E. coli* BL21 (DE3) cells harbouring the recombinant plasmid were used in the expression study. The entire transformation reaction was then inoculated into 10 ml LB broth containing 100 µg/ml ampicillin and incubated overnight at 37^ο^C with shaking at 200 rpm. An aliquot of the overnight cell culture was added into another tube of LB medium (containing 100 µg/ml ampicillin) and incubated at 37°C with shaking (200 rpm). Once an optical density at 600 nm of the cultures reached 0.5–0.8 (mid log), cells were induced with 0.25 mM isopropyl thiogalactoside (IPTG).

### Purification of protein

Recombinant protein was purified by Ni–NTA (nickel–nitrilotriacetic acid) affinity chromatography (Qiagen) and molecular sieve chromatography respectively. After induction, recombinant cells (0.5 g) were lysed by gentle stirring in lysis buffer (8 M Urea; 0.1 M NaH_2_PO_4_; 0.01 M Tris–Cl; pH 8.0), centrifuged at 10,000 *g* for 30 min. Lysate was again mixed with 50% Ni–NTA slurry (4∶1) and kept at 4°C for 1 h. Ni–NTA slurry was loaded on the column and finally the purified protein was eluted using elution buffer (8 M Urea; 0.1 M NaH_2_PO_4_; 0.01 M Tris–Cl; pH 4.5) after washing with wash buffer (8 M Urea; 0.1 M NaH_2_PO_4_; 0.01 M Tris–Cl; pH 6.3 and 5.9). Additional purification of the protein was achieved by using the Sephacryl S-200 HR column. The affinity purified fraction was dialysed in the buffer 20 mM Tris-HCl (pH-8.0)-0.1 M NaCl-10 mM EDTA-0.4 % CHAPS and then applied to Sephacryl S-200 HR column pre-equilibrated with the same buffer. The expression and purity of the recombinant protein was confirmed by running on 10% SDS–PAGE [22]. The lipopolysaccharide in the purified protein was then removed as described previously [18]. The recombinant gel filtration purified protein was passed through Detoxi-Gel endotoxin–removing resin and S3Δ peptide affinity gel columns respectively. Absence of traces of LPS in the purified protein was confirmed by the *Limulus* amoebocyte lysate chromogenic assay with Kinetic-QCL® (Lonza). The concentration of protein was estimated by Bradford method [23]. Purified recombinant OmpA was then dialysed in 20 mM Tris-HCl-0.4% CHAPS-10 mM EDTA and finally stored at −20°C for further use. This recombinant lipopolysaccharide free protein was employed in all the experimental methods described here.

### Raising murine antisera

BALB/c mice were immunized intraperitoneally with the recombinant OmpA emulsified with 2 volumes of Freund’s complete adjuvant (Gibco). Two booster immunizations were followed at an interval of 10 days and the sera were collected by puncturing of the supraorbital plexus.

### Collection of human sera

Sera were collected from blood by standard laboratory procedure of normal human volunteers, and at day 21 (convalescent) of adult, male patients recovering from shigellosis after admission to the Infectious Diseases and Beliaghata General Hospital, Beliaghata, Kolkata-700 010.

### Immunoblotting

The whole-cell antigens of *Shigella flexneri* 2a, *S. boydii*, *S. sonnei*, *S.* dysenteriae type 1, enteropathogenic *E. coli* O115, the recombinant induced *E. coli* and the recombinant his-tag OmpA were separated by SDS-PAGE and transferred electrophoretically to nitrocellulose membrane (Transblot; Bio-Rad Laboratories, USA) under wet transfer condition as described previously [17]. Free sites on nitrocellulose strips were then blocked with TBS-5% (w/v) defatted milk (Bio-Rad) powder for overnight at 4°C. They were then incubated sequentially with mouse antiserum (1∶1000, v/v), raised against the recombinant OmpA of *Shigella flexneri* 2a [For cell-lysate], patients sera and mouse antiserum produced by challenged with live whole *S. flexneri* 2a [For OmpA] respectively in TTBS (containing TBS with 0.1% Tween-20 and 0.5% non fat dry milk) at room temperature for 2 h and alkaline phosphatase conjugated goat anti-mouse IgG (1∶2000, v/v [Jackson]) in TBS (containing 0.5% non-fat dry milk) for 1 h at room temperature. Nonspecifically bound proteins were removed by washing nitrocellulose strips with TTBS thrice between two incubation steps. Immunoreactive bands were visualized by color development with alkaline phosphatase using 5-bromo-4-chloro-3 indolyl phosphate (BCIP, 3.75 mg in 250 µl of 100% DMF [Sigma]) and nitroblue tetrazolium (NBT, 7.5 mg in 250 µl of 70% DMF [Sigma]) in carbonate buffer, pH-9.8, containing 0.1 M NaHCO_3_ and 1 mM MgCl_2_.

### Immunogenecity and protective capacity of the over expressed his-tag OmpA

The ability of the expressed his-tag OmpA to promote an immune response was tested in BALB/c mice. Seven-week-old BALB/c female mice weighting approximately 25 gm were sedated by intramuscular injection of a mixture of 0.3 mg of xylazine hydrochloride and 1.0 mg of ketamine hydrochloride in 50 µl of saline. Each mouse was immunized intranasally with 3 µg of recombinant his-tag OmpA or his-tag removed OmpA of *Shigella flexneri* 2a on day 0, 14, and 28. A total antigen volume of 25 µl was delivered in five to six small drops applied to the external nares with a micropipette. Control animals were inoculated intranasally with 0.9% saline.

Three weeks (day 49) after the final immunization, all mice were challenged intranasally with a lethal dose of *S. flexneri* 2a (1×10^7^ CFU/30 µl) as described for the mouse lung model [24]. The mouse challenge dose was prepared from a frozen lot of *S. flexneri* 2a that had been harvested during the log phase of growth, which is the time of optimal invasiveness for shigellae, and then stored in liquid nitrogen. Mice were monitored for weight loss, lethargy, fur ruffling and death for 14 days after challenge. A total of 42 mice were taken for each set of experiment. One mouse was used as unimmunized control. All mice were then divided into two groups. 20 mice were taken as saline immunized control and 21 mice were used for protein immunization purpose with the recombinant protein. In the protein immunized group except one mice (for histopathological study) all mice (20 mice) were challenged three weeks after final immunization (day 49). Among the 20 mice, 16 were kept for observation till fourteen days after treatment (day 63) and 4 mice were sacrificed within 24 h of challenged for histopathology and cytokine assay. From the saline immunized control group of mice [20], all mice were challenged and among these 20 mice, 16 mice were kept for observation till fourteen days after treatment (day 63) and 4 mice were sacrificed within 24 h of challenged for histopathology and cytokine assay.

### Sampling of immune response

For antibody detection blood was taken by tail bleed from all mice on days 0, 28, 42, and 63. For cytokine studies, lavage fluid was collected within 24 h of postchallenge. Pulmonary lavage was performed by inflating the lungs with cold RPMI 1640 and by withdrawing the fluid through trachea. Lavage fluids were maintained at 4°C and then centrifuged to remove the cellular debris. Sera and lavage fluids were stored at −70°C until used for antibody and cytokine studies. Intestinal lavage (IL) fluid from mice was collected as described previously [25]. Splenocytes for ASC were harvested from the spleen of immunized mice by mincing and teasing the tissue. Spleens were strained over 70 µm nylon cell strainers. The resulting suspension of splenocytes was layered over Histopaque® and mononuclear cells were isolated via differential centrifugation. The cells were then washed in RPMI 1640 with 5 U/ml penicillin G, 5 µg/ml streptomycin, and 0.1% gentamycin and then used in enzyme-linked immunospot (ELISpot) assay.

### Enzyme-linked immunosorbent assay (ELISA)

Antigen-specific antibody responses in sera and mucosal samples were assessed by ELISA as previously described [26]. Test wells of 96-well microtitre plate (Corning, USA) were coated with 100 µl of the recombinant his-tag protein or his-tag removed protein sample diluted in PBS (2 µg/ml) by overnight incubation at 4°C; blank wells were coated with buffer alone. Unbound antigen was decanted, washed thrice with PBS (pH 7.2) -0.05% (v/v) Tween 20. Free sites were blocked by addition of 250 µl of 2% BSA in PBS and incubated for 2 h at room temperature (RT). Two-fold serially diluted samples (starting from 1∶32 for serum; 1∶2 for lung lavage) were applied to plates and incubated at RT for 2 h. After incubation, the wells were washed with PBS containing 0.05% (v/v) Tween 20. Bound antibodies were detected by incubation with HRP-conjugated goat anti-mouse IgG Ab or goat anti-mouse IgA Ab. The wells were washed, colour was developed with 100 µl of TMB (3, 3′, 5, 5′-tetramethylbenzidine) solution (Pierce) and then kept for 15 min at RT. The reaction was stopped by the addition of 50 µl of 1 N H_2_SO_4_ (Sigma). Absorbance was read by a microplate reader (BIO-RAD). Endpoint titers of OmpA-specific Abs were expressed as the reciprocal log_2_ of the last dilution giving an optical density at 450 nm of 0.1 or greater.

### ELISpot assay for detection of numbers of antibody-secreting cells (ASCs)

Spleen cells were suspended to a density of 2.5 ×10^6^ /ml in complete medium consisting of RPMI 1640 supplemented with 5 U/ml penicillinG, 5 µg/ml streptomycin, 0.1% gentamycin, 10 % fetal bovine serum (Gibco). OmpA specific antibody-secreting cells were quantitated by the method of Ndungu et al., [27] with modifications. Briefly, the 96-well nitrocellulose bottom microplates (BD Falcon) were coated with 2 µg/ml of the OmpA in phosphate-buffered saline (PBS) and incubated at 4°C overnight. One-hundredmicroliter volumes of the cells were allowed to incubate for 4 h in 5 % CO_2_ at 37°C in the coated wells. The plates were washed and then the wells were incubated with HRP-conjugated goat anti-mouse IgG or IgA overnight at 4°C. Finally the detection was carried out by the addition of the substrate reagent (TMB) in each well. Spot-forming cells were counted under a stereomicroscope (Olympus). Results were recorded as the number of ASC per 10^6^ splenocyte.

### Histopathological analysis of tissue samples

Twenty-four hours after bacterial infection, the lung was perfused with PBS through the abdominal aorta, removed and fixed in 4% formaldehyde for 1 h at 4°C. The tissues were dehydrated by gradually soaking in alcohol and xylene and then embedded in paraffin. The paraffin-embedded specimens were cut into 5 µm sections and stained with hematoxylin and eosin. The sections were viewed using a digital light microscope (Olympus).

### Cytokine ELISA

TNF-α, IL-6, MIP-2, and IFN-γ in lung lavage were measured in sandwich ELISA according to the manufacturer’s instruction (R & D System).

### Statistical analysis

The statistical significance of difference between the test groups was analysed by Student’s t-test (two-tailed) using SPSS 7.5 software. Results were expressed as the mean ± standard error of the mean (S.E.M) where applicable, of three independent experiments. Statistical significance was assumed at p<0.05.

## Results

### Expression and characterization of the recombinant his-tag OmpA

**Figure 1:**
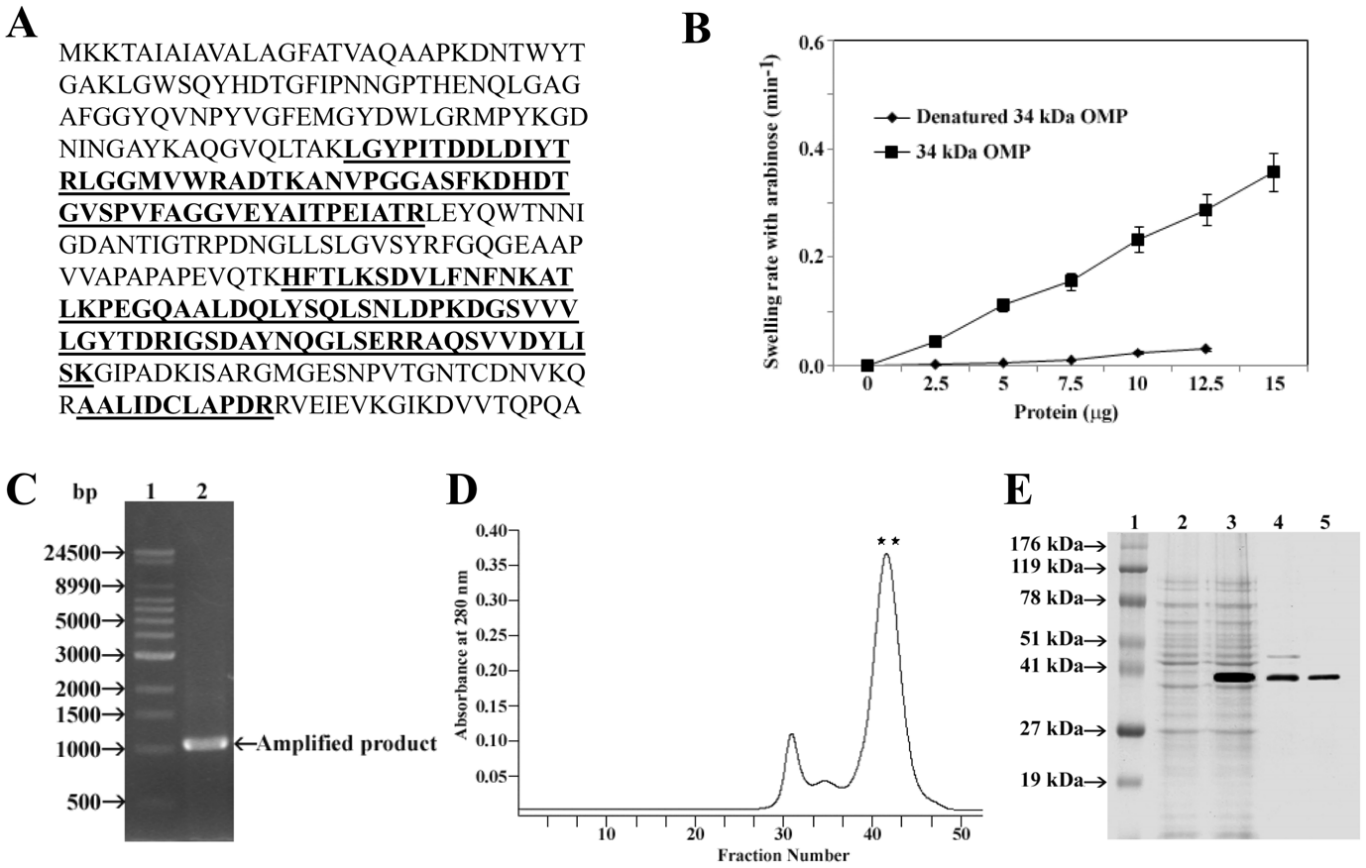
Sequencing and liposome swelling assay of 34 kDa OMP and its expression analysis. **A:** MALDI-TOF MS of purified 34 kDa OMP. The amino acid sequence of the 34 kDa OMP of *S. flexneri* 2a 2457T is given here. Peptides confirmed by MS/MS sequencing are shown in bold. **B:** Swelling rate of liposomes reconstituted with *S. flexneri* 2a 34 kDa OMP. Different concentration of purified 34 kDa OMP of *S. flexneri* 2a or denatured 34 kDa OMP by boiling in 1% SDS at 100°C were reconstituted with 3.1 µmol of egg phosphatidylcholine and 0.2 µmol of dicetyl phosphate. Then, 13 µ1 of the proteoliposome suspensions were diluted into 0.6 ml of iso-osmotic L-arabinose, and the initial rates of OD_400_ decrease were measured. Each point is an average of three data. **C:** PCR amplification of the *ompA* gene. Lane 1, molecular weight marker (Supermix DNA ladder); lane 2, *Shigella flexneri 2a*. **D:** Elution profile (A_280_) of the Ni-NTA purified fraction on a Sephacryl S-200 HR Column. The Ni-NTA purified fraction was applied to a column (1.8 cm by 48 cm) packed with Sephacryl S200 HR, which had been equilibrated with a buffer (20 mm Tris-HCl [pH-8], 100 mM NaCl, 10 mM EDTA and 0.4 % CHAPS). The flow rate was 0.5 ml/min. ** indicates the elution of the purified recombinant his-tag OmpA. **E:** Expression of recombinant his-tag OmpA. Lane 1, Lonza ProSieve® Color Protein molecular weight marker; lane 2, recombinant BL21 Star™(DE3) *E. Coli* without IPTG; lane 3, recombinant BL21 Star™(DE3) *E. Coli* with 0.25 mM IPTG; lane 4, Ni-NTA purified fraction; lane 5, Sephacryl S-200 HR purified fraction.

MALDI-TOF MS analysis of the 34 kDa OMP (Fig. 1A) identified it as the outer membrane protein A (OmpA) of *Shigella flexneri* 2a (sequence Identity, 65% with an e score of 4e-68) by using Mascot (www.matrixscience.com). To confirm that the 34 kDa OMP of *S. flexneri* 2a is OmpA, the pore-forming activity of 34 kDa OMP from *S. flexneri* 2a was determined by reconstitution into proteoliposomes and by the osmotic swelling of these vesicles detected by following the optical density of the suspension. As seen in Fig. 1B, the permeability of liposomes toward L-arabinose was proportional to the amount of 34 kDa OMP added. From BLAST analysis the respective gene of the OmpA of *S. flexneri* 2a was identified (Accession No. NP_836665) and primer sequences were designed from the conserved domains of the *ompA* gene of *S. flexneri* 2a. By using the primers, a DNA fragment with a size of 1,047 bp was amplified (Fig. 1C) and sequenced. A sequence analysis of the amplified fragment revealed the highest homology to the *ompA* gene from *S. flexneri* 2a. The amplicon was then cloned into pET100/D-TOPO® linearized vector using One Shot® TOP10 *E. coli* as host cells. After ampicillin selection, colonies were screened for the insert using the same primers and PCR conditions. Finally the vector was introduced into *E. coli* BL21 (DE3) cells for protein overexpression and purification. The OmpA was expressed in BL21 Star™ (DE3) *E. coli* with N-terminal 6× histidine fusion. Following gentle purification (Fig. 1D), the expressed protein obtain was estimated to be approximately 37 kDa by 10% SDS-PAGE (Fig. 1E) with a yield of 2.5 mg/litter of culture. The observed size of the recombinant protein was slightly larger than the calculated ones due to the presence of hexa-histidine.

**Figure 2:**
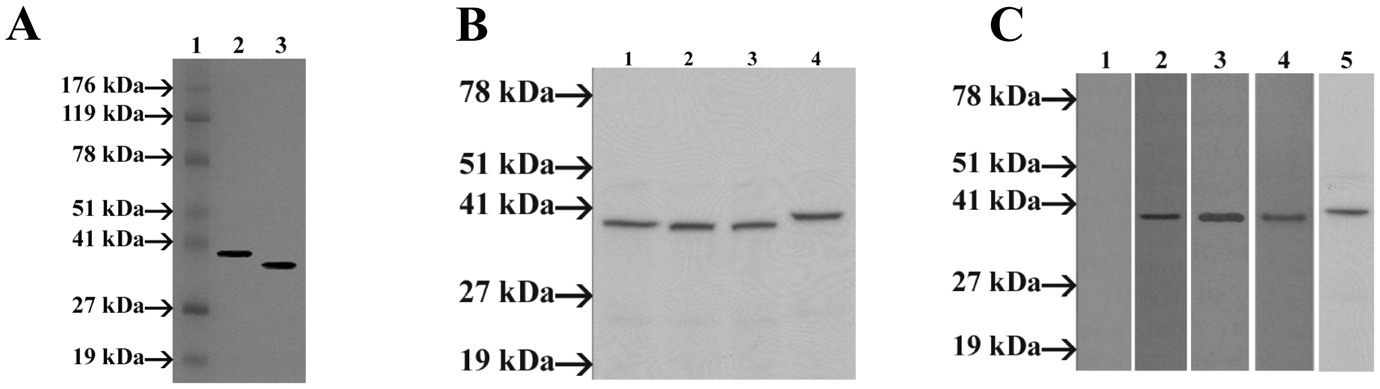
Characterization of the recombinant his-tag OmpA. **A:** Immunoblotting analysis of the OmpA using mouse polyclonal antibody raised against the recombinant OmpA. Lane 1, Lonza ProSieve® Color Protein molecular weight marker; lane 2, recombinant BL21 Star™(DE3) *E. Coli* with 0.25 mM IPTG; lane 3, *S. flexneri 2a* whole-cell lysate. **B:** Immunoblot analysis of whole-cell preparations of *Shigella* spp., with *S. flexneri* 2a OmpA murine antiserum. *S. flexneri* 2a OmpA antiserum was used to probe the whole-cell antigen of *S. flexneri* 2a (lane 1), *S. boydii* (lane 2), *S. sonnei* (lane 3), *S. dysenteriae* type 1 (lane 4). Molecular mass standards are noted on the left. C: Western blot analysis of recombinant his-tag OmpA of *S. flexneri* 2a with convalescent-phase serum of shigellosis patients. OmpA of *S. flexneri* 2a was electrophoresed and transferred to nitrocellulose membrane and finally probed with 21-day-old convalescent sera obtained from two patients (lane 1 to 3). Lane 1. Normal serum, Lane 2. Patient-1 serum, Lane 3. Patient-2 serum. Western blot analysis of recombinant OmpA of *S. flexneri* 2a with antisera from virulent *Shigella flexneri* 2a challenged mice (lane 4) and immunoblot analysis of recombinant his-tag OmpA antiserum with the whole-cell lysate of enteropathogenic *E. coli* O115 (lane 5). Molecular mass standards are noted on the left (Lonza ProSieve® Color Protein molecular weight marker).

Murine antiserum raised against the recombinant his-tag OmpA recognized the whole-cell lysates of *S. flexneri* 2a and recombinant *E. coli* (induced) as the antigen in an immunoblot analysis (Fig. 2A). It was found that OmpA antiserum cross-reacted with the whole cell lysates of *S. boydii*, *S. sonnei*, and *S. dysenteriae* type 1 (Fig. 2B) as well as with the whole cell antigen of enteropathogenic *E. coli* O115 (Fig. 2C, lane 5). Therefore, our study indicates that OmpA of *S. flexneri* 2a may play important role in cross protection study. The recombinant protein also showed reactivity with mouse antisera produced by challenged with whole cell virulent *S. flexneri* 2a (method not described here), suggesting the strong immunogenecity of the recombinant OmpA (Fig. 2C, lane 4). This also proved that the antigenicity of the protein is maintained.

### Prevalence of antibodies to the OmpA in patients with shigellosis

To determine whether sera from patients with shigellosis contain antibodies to the OmpA, immunoblot analysis with the recombinant his-tag OmpA preparation as the antigen was performed. Convalescent sera (1∶1000), which were collected from patients at 3 weeks after *S. flexneri* 2a infection, reacted specifically and intensely with the recombinant OmpA of *S. flexneri* 2a (Fig. 2C, lane 1–3).

### Immunogenecity and safety of expressed his-tag OmpA in mice

**Figure 3:**
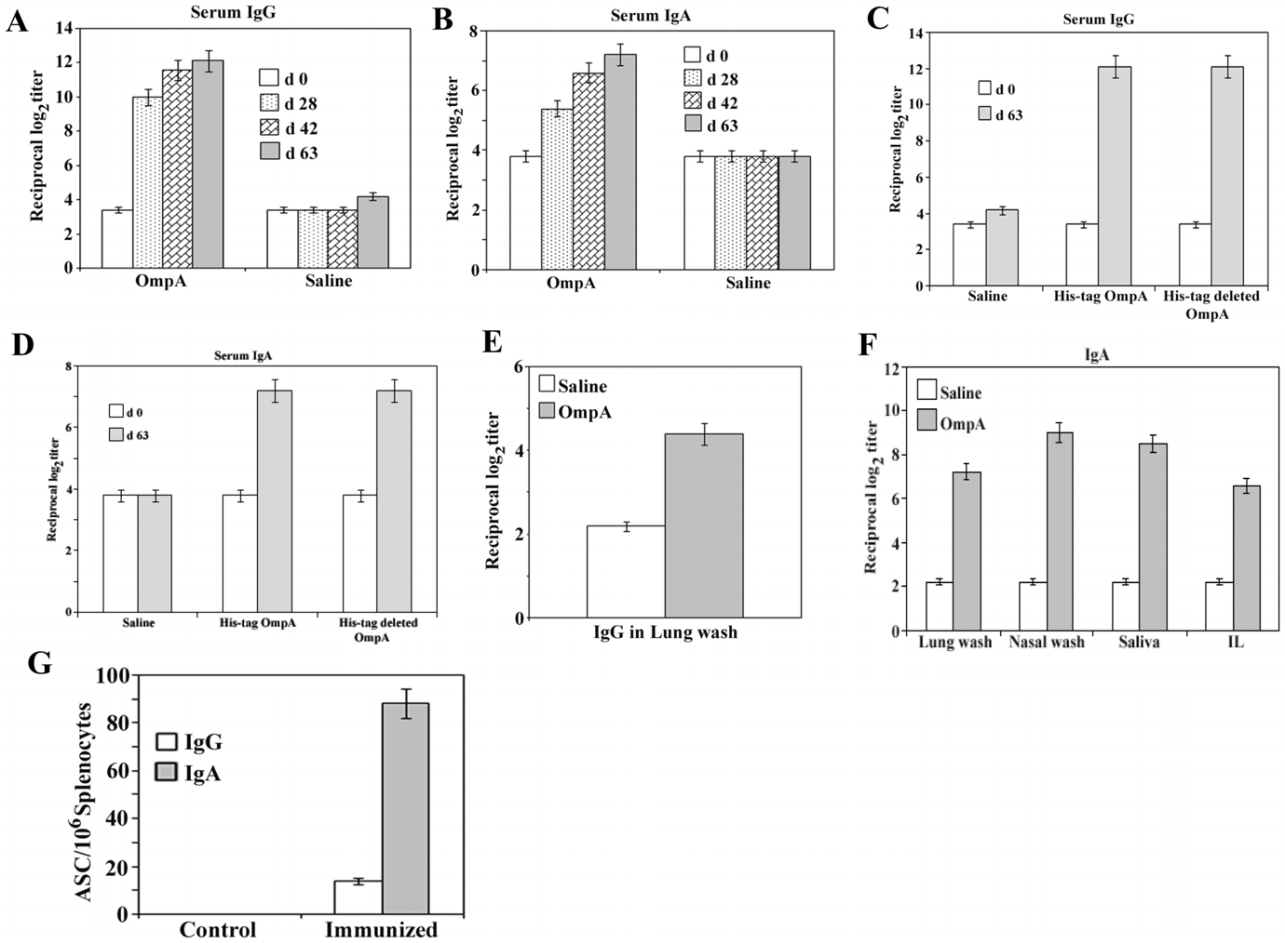
Recombinant OmpA elicits strong mucosal and systemic immune responses to induce protective immunity. **A:** Serum IgG response in mice immunized with recombinant his-tag OmpA followed by lethal challenge with *S. flexneri* 2a. Mice were intranasally immunized with recombinant his-tag OmpA or saline on days 0, 14, 28. Blood collected on days 0, 28, 42 and 2 weeks after challenge (day 63) were analysed by ELISA for antibody levels to recombinant OmpA. The values represent the mean endpoint (log_2_) antibody titer ± S.E.M. in each experimental group. The experiment was repeated three times with similar results, p<0.05. **B:** Recombinant his-tag OmpA induced serum IgA response in mice immunized on days 0, 14, 28 by the intranasal routes. Blood collected on days 0, 28, 42 and 2 weeks after challenge (day 63) were analysed by ELISA for IgA levels to recombinant OmpA. The values represent the mean endpoint (log_2_) antibody titer ± S.E.M. in each experimental group. The experiment was repeated three times with similar results, p<0.05. **C:** Comparison of serum IgG response in mice immunized with recombinant his-tag OmpA or his-tag removed OmpA of *S. flexneri* 2a followed by lethal challenge with *S. flexneri* 2a on days 0 and 63. The values represent the mean endpoint (log_2_) antibody titer ± S.E.M. in each experimental group. The experiment was repeated three times with similar results, p<0.05. **D:** Evaluation of serum IgA response in mice immunized with recombinant his-tag OmpA or his-tag deleted OmpA of *S. flexneri* 2a followed by lethal challenge with *S. flexneri* 2a on days 0 and 63. The values represent the mean endpoint (log_2_) antibody titer ± S.E.M. in each experimental group. The experiment was repeated three times with similar results, p<0.05. **E:** IgG levels in lung wash. Each group of mice was immunized intranasally with saline or recombinant his-tag OmpA. Following the final immunization, lung washes were collected and then analyzed for recombinant his-tag OmpA specific IgG by ELISA. The values shown are the mean endpoint (log_2_) antibody titer ± S.E.M. in each experimental group. The experiment was repeated three times with similar results, p<0.05. **F:** Intranasal immunization with the recombinant his-tag OmpA induces systemic and mucosal immune response in mice. Groups of mice were immunized with saline or recombinant OmpA by the intranasal routes. After final immunization, lung wash, nasal wash, saliva and intestinal lavage (IL) fluid were collected and then the levels of protein specific IgA were measured by ELISA. The values represent the mean endpoint (log_2_) antibody titer ± S.E.M. in each experimental group. The experiment was repeated three times with similar results, p<0.05. **G:** Analysis of recombinant his-tag OmpA specific ASCs in mice immunized nasally with the protein. Seven days after the last nasal immunization, mononuclear cells isolated from spleen were examined using the recombinant protein-specific ELISpot assay to determine the numbers of IgG, and IgA ASCs. The results represent the mean values ± S.E.M. of three independent experiments, p<0.05.

To ascertain the immunogenecity and safety of the recombinant protein in vivo, groups of mice were immunized with the protein by the intranasal route. Death or visible side effects due to toxicity (such as ruffled fur or lethargy) did not occur in mice immunized with 3 µg of OmpA of *S. flexneri* 2a on days 0, 14, and 28. Intranasal immunization of mice with the OmpA developed pronounced serum IgG and IgA antibody responses to the homologous antigen (Fig. 3A and 3B). Furthermore, no significant difference was observed in serum IgG and IgA antibody responses among mice immunized with recombinant his-tag OmpA or his-tag removed OmpA (Fig. 3C and 3D), signifying that the his-tag is not playing any important role in the measured immune response to OmpA. The IgG titer in the lung lavage fluid of immunized mice was significantly higher than the control mice (Fig. 3E). It was also found that nasal administration of the recombinant his-tag OmpA elicited significantly high levels of OmpA antigen specific IgA antibody in the mucosal secretions, i.e., lung wash, nasal wash, saliva, and intestinal lavage (Fig. 3F). Moreover, trafficking of antibody-secreting B-cell lymphoblasts (ASC) to distal tissues after final immunization was measured by using the mononuclear cells isolated from spleen. Intranasal immunization of OmpA induced significantly higher numbers of OmpA antigen specific IgA ASCs in the spleen (Fig. 3G). These data demonstrate that the protein effectively induces both systemic and mucosal immune responses in mice.

### Protective efficacy of the overexpressed his-tag OmpA of *S. flexneri* 2a

**Figure 4:**
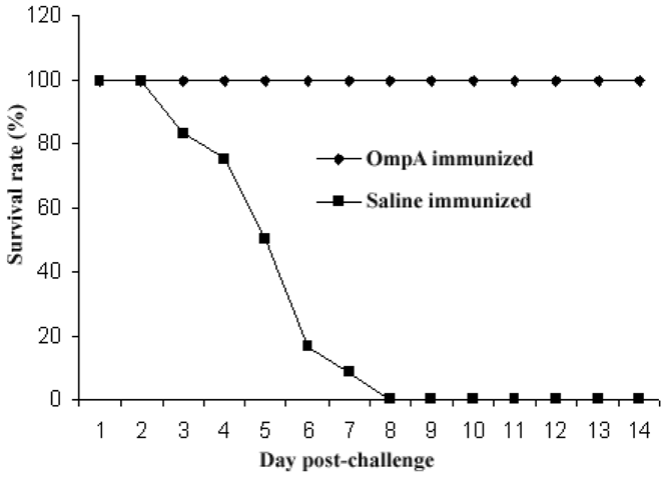
Mice survival rate after immunization with recombinant his-tag OmpA. Two groups of 20 mice were immunized with normal saline or OmpA. Mice were immunized intranasally at 2-week intervals with 3 µg of OmpA in each immunization. Three weeks after the final immunization, all animals were intranasally challenged with 1×10^7^ CFU of *S. flexneri* 2a. Figure representing the result for 16 mice from each group, as 4 mice from each group were sacrificed within 24 h after challenge for histopathology and cytokine assay. Percent survivors are plotted for each of 14 days postchallenge. *P* value, calculated by the Fisher exact test and is <0.001.

Three weeks after the final immunization (day 49), all mice were challenged with 1×10^7^ CFU virulent *S. flexneri* 2a into their nostrils. Significantly high (100%) level of protection against lethal challenge was achieved in mice immunized with the protein (Table 1). The saline treated control mice showed ruffled fur, gradually become lethargic and finally death occurred within day 3 to 8 (Fig. 4). Among the saline treated control mice three mice were survived and recovered gradually with little symptoms. Animals received OmpA, initially (over 2 days) lost weight upon challenge (19%) and then began to recover and gain 60% of their pre-challenged weight by day 7. All the OmpA immunized mice finally return to normal over the 14 day observation phase.

**Table 1:** Protective efficacy of the recombinant his-tag OmpA in the mouse pulmonary infection model (data representing 16 mice from each group).

Antigen^a^	Challenge^b^	#Survivors/total^c^	% Protection^d^	P-value^c^
Recombinant his-tag OmpA	*S. flexneri* 2a	16/16	100	<0.001
0.9% saline	*S. flexneri* 2a	3^e^/16	0	-

a The protective efficacy of the recombinant his-tag OmpA was evaluated with the mouse lethal lung model. Groups of 20 mice were immunized intranasally with the recombinant his-tag OmpA or normal saline. Table showing the data for 16 mice from each group, as 4 mice from each group were sacrificed within 24 h after challenge for histopathology and cytokine assay.

b Three weeks after the final immunization, mice were challenged with virulent *S. flexneri* 2a (N.Y-962/92).

c Deaths were recorded daily for 14 days after challenge.

d % of protection was calculated by the following formula: [(percent deaths of controls) - (percent deaths of immunized mice)]/[percent deaths of controls] ×100.

c P values were determined by the Fisher’s exact test.

e Three saline immunized control mice did not die after challenge.

### Histology of mouse lung tissue after *S. flexneri* 2a challenge

**Figure 5:**
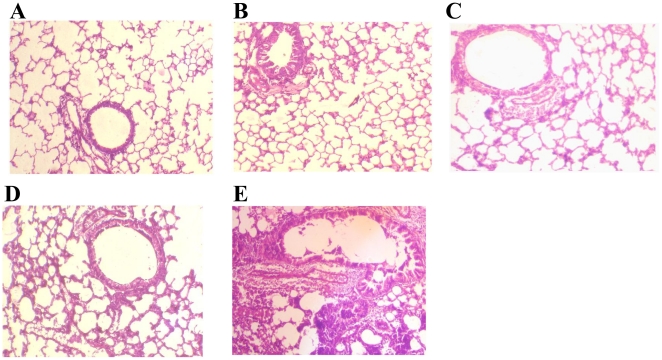
Hematoxylin and eosin-stained light micrographs of histological sections from mice lungs (Magnifications: X10). Lungs from nonimmunized mice (**A**), from saline immunized mice (**B**) and from intranasal OmpA immunized mice (**C**) showing normal airspaces, interstitium, and bronchioles. **D**: Intranasal OmpA immunized mouse lung tissue, 24 h after intranasal challenge with a lethal dose of *S. flexneri* 2a (1×10^7^ CFU/30 µl) showing mild infiltration of neutrophils and other mononuclear cells. **E**: Lung from saline immunized mice, 24 h after intranasal challenge with a lethal dose of *S. flexneri* 2a N.Y-962/92 showing severe pneumonia with vascular congestion, peribronchial and perivascular accumulation of inflammatory cells, including polymorphonuclear neutrophils and mononuclear cells.

Lungs from nonimmunized mice, from saline immunized mice and from intranasal recombinant his-tag OmpA immunized mice illustrated normal lung morphology (Fig. 5A, 5B, and 5C). The recombinant his-tag OmpA immunized mice also showed normal lung architecture, with mild infiltration of neutrophil and mononuclear cells (Fig. 5D) after challenge with virulent *S. flexneri* 2a. The mice immunized with saline demonstrated significant perivascular and peribronchial inflammation with neutrophil and mononuclear cells, characteristic of severe pneumonia (Fig. 5E).

### Cytokine levels in the lung lavage

**Figure 6:**
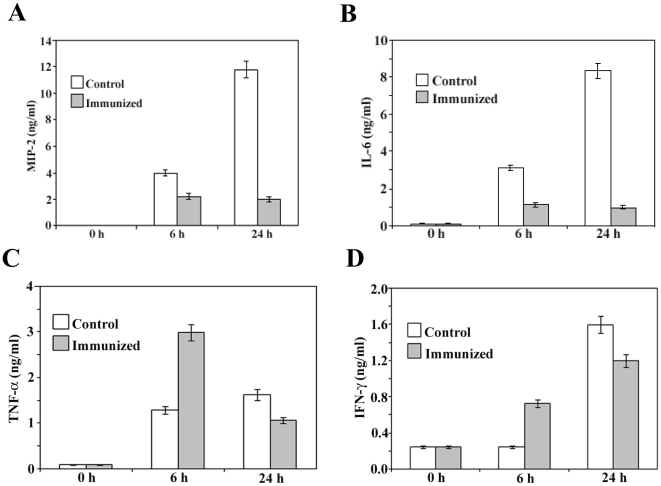
Time course of cytokine expression following lethal challenge of mice that had been previously immunized with recombinant protein or saline (control). MIP-2 (A), IL-6 (B), TNF-α (C), and IFN-γ (D) were assayed by ELISA with undiluted lung lavage samples. Data represent mean ± S.E.M. of three independent experiments with similar results, p<0.05.

In the present study the immunogenecity and protective efficacy of the recombinant his-tag OmpA of *S. flexneri* 2a was executed in mouse pulmonary pneumonia model. The lung is a lymphoid organ with antigen-presenting cells, T helper and suppresser lymphocytes, and B lymphocytes [28]. To gain deeper insight into the biological mechanisms responsible for the superior vaccine efficacy of OmpA, immune responses in vivo were analyzed in the early and latter infectious stages of lung infection. Consequently, lung lavage fluid was collected before challenge (0 h) and 6, and 24 h after intranasal challenge of both immunized and control mice with lethal dose of virulent *S. flexneri* 2a. The levels of proinflammatory cytokines, IL-6, TNF-α, IFN-γ and MIP-2 in the lavage fluid were then measured. 6 h after challenge, the levels of MIP-2 (Fig. 6A), a key murine PMN attractant, were found to be raised above base line in the lungs of both control and immunized mice, with a significant more elevation in the former ones. The level of other proinflammatory cytokine, IL-6 (Fig. 6B) was also significantly increased with time in the lungs of control mice, but lesser amounts were produced in the immunized mice. 6 h after challenge, TNF-α responses (Fig. 6C) were detected in both control and immunized mice, with higher values in the latter animals. The levels of TNF-α began to decline after 24 h in the immunized mice, while levels in control mice increased. IFN-γ was detectable in immunized and control mice 6 h after challenge (Fig. 6D), with significant more elevation in immunized mice than those in control. The levels of this cytokine began to rise in control mice within 24 h than the immunized one.

## Discussion

Shigellosis continues to be a major health concern worldwide, occurring predominantly in children younger than five years of age in developing countries [1]. In view of increasing antibiotic resistance, vaccination appears to be the only rational prophylactic approach to control shigellosis. Previously 34 kDa OMP of *Shigella flexnreri* 2a has been purified and characterized as a potent immunostimulant [17], [18]. It has been reported earlier that the amino terminal sequence of the purified protein does not match with any known membrane protein of *S. flexneri* 2a [17]. Therefore, MALDI-TOF MS of the purified 34 kDa OMP has been performed to predict the amino acid sequence of the full-length protein, which exhibits considerable sequence homology (Identity 65%) to the OmpA of *S. flexneri* 2a. In this communication it has been observed that the purified 34 kDa OMP of *S. flexneri* 2a produces diffusion channels in liposome swelling assay, confirms the identity of the protein as OmpA. In the present study the OmpA of *S. flexneri* 2a has also been successfully cloned, over expressed and its safety and protective activity as vaccine candidate in mice has been assessed for the first time. Immunization with the recombinant his-tag OmpA does not induce severe inflammatory responses, as manifested by ruffled fur, lethargy and death of the vaccinated mice.

Outer membrane protein A (OmpA), which is found in many gram-negative bacteria and which was studied most extensively in *E. coli*, is necessary for maintenance of structural integrity of cell envelopes [29]. OmpA is essential for bacterial survival and pathogenesis [30]. It is involved in bacterial conjugation [31], in bacterial attachment, as receptors for certain bacteriophages, and in porin activity. OmpA is also known to be associated with the pathogenesis and plays a key role during the initial processes of bacterial adhesion and invasion [32]. OmpA-deficient *E. coli* mutants grow normally but exhibit attenuated virulence, invasive capacity, and resistance to serum bactericidal activity [33]. OmpA is also known to stimulate a strong antibody response [34]. Furthermore, a number of studies reveal the strong immunogenecity of OmpA. It has been observed that recombinant OmpA of *Klebsiella pneumoniae* binds to and activates both macrophages and dendritic cells [35]. Dendritic cells activation and cytokines production have also been found by OmpA of *E. coli* [36]. This property contributes to explain its immunogenecity. Recently Dumetz et al., demonstrates that the OmpA of *Flavobacterium psychrophilum* as a possible candidate for the development of subunit vaccine [37]. In addition, several other studies have illustrated OmpA as a potential vaccine candidate [38]–[40].

In the present study protective efficacy of the recombinant protein has been evaluated in mouse pulmonary model. The mouse lung model has been chosen, as the pathology of pulmonary lesions closely mirroring the colitis that characterizes human shigellosis [41]. Our study clearly demonstrates that intranasal immunization of mice with the recombinant his-tag OmpA induces strong protective immunity (100%) against a lethal challenge of *S. flexneri* 2a i.e. elicits strong protective immunity to pulmonary pneumonia as well as robust levels of systemic and mucosal immunity. Whether the recombinant his-tag OmpA of *S. flexneri* 2a shows heterologous protective efficacy in other *Shigella* spp., is of great interest and should be evaluated in future studies.

In order to access the immunogenicity of the recombinant his-tag OmpA, protein specific IgG and IgA responses have been measured. Immunization of mice result in significantly enhanced protein specific serum and pulmonary IgG antibody responses. The production of IgA has also been assessed, the hallmark of mucosal immuneresponsiveness, in serum and in various mucosal samples. Nasal administration of the protein induces significantly higher levels of protein specific IgA production in serum and in all of the mucosal samples tested. These results signify that the OmpA is an efficacious mucosal immunogen for the induction of antigen-specific systemic IgG and mucosal IgA productions. Furthermore, trafficking antibody-secreting cells (ASCs) in the spleen has been monitored to reflect the antibody levels in the lungs. Our result shows that IgA-secreting cells, which have been stimulated as a result of immunization, travel through the spleen and colonize the common mucosal system. On the other hand, comparatively low levels of IgG-secreting cells have been detected in the spleen although high levels of IgG have been detectable in lung lavages. It may be possible that IgG ASC traffic predominately through peripheral lymphoid tissues following immunization. IgA is a critical component of the mucosal immune response and its secretion upon mucosal immunization provides an important first line of defense against invasion of bacterial and viral pathogens [42], [43].

In this study, we also demonstrate that antibody to the recombinant his-tag OmpA is present in sera from patients with shigellosis. The presence of antibody to the OmpA in convalescent sera suggests that this OMP is potentially antigenic and elicits an antibody response in shigellosis patients. The recombinant protein also reacted strongly with antisera produced by challenge in mice with live whole cell *S. flexneri* 2a, again indicating the strong immunogenecity of the protein.

Histology and time kinetic study of the local cytokines productions have also been evaluated to indicate the immune status of the animals, at the time of challenge after immunization. The cytokine profile of the innate immune response can influence the profile of the subsequent specific immune response linked to the development of immunity to shigellosis. Significantly more elevation of MIP2 has been observed in the control mice than the immunized mice 24 h after challenge correlates with histological analysis showing extensive infiltration of neutrophils and mononuclear cells. Massive recruitment of neutrophils has been found to the site of *Shigella* infection [44]. On the contrary the mild infiltration of PMN in the immunized mice could play crucial role in the protective immunity by enhancing bacterial clearance [45]. A prolong elevation of TNF-α and IL-6 levels have been observed in the control mice compared with that in the immunized mice. It is known that high and sustained local intestinal levels of TNF-α and IL-6 are present during established shigellosis [46], [47]. In the immunized mice the production of TNF-α and IL-6 have been found to peak at 6 h after challenge and then decreased thereafter. TNF-α and IL-6 are important for the early innate immune response in bacterial infection [48]. Moreover, IL-6 activities are critical for resolving innate immunity and promoting acquired immune responses [49]. This transient rise of TNF-α in immunized mice could play important role in the recruitment of antigen presenting cells (macrophages) and upregulation of antigen presentation as well as reflecting that Th1 response playing prominent role in the protective immunity against lethal *S. flexneri* challenge. This result is consistent with the fact that *Shigella* is an intracellular pathogen. Whereas the sustained elevation of TNF-α and IL-6 in saline treated mice corroborates with the massive tissue damage by the infiltrating monocytes or macrophages, an important characteristic of shigellosis, and ultimate death. The level of IFN-γ has also been found to increase in the lungs of both immunized and control mice, with an earlier response in the former animals. The rapid increase in IFN-γ production observed in lungs collected post challenge suggests that Th1 lymphocyte activation in shigellosis may stimulate macrophages leading to the elimination of phagocytosed *Shigella* organisms. In addition to promoting Th1 responses, IFN-γ serves as an effector molecule by preventing *Shigella* invasion of eukaryotic cells [50]. It is therefore tempting to speculate that this cell-mediated response, in combination with secretory IgA, may account for the robust level of protection against virulent *S. flexneri* 2a infection in the immunized mice.

So, our current study has clearly been identified OmpA as a potent mucosal subunit vaccine candidate. The high levels of protein-specific IgG and IgA Ab as well as of ASCs stimulated by intranasal administration suggest that mucosal immunization with recombinant his-tag OmpA is an effective regimen for inducing both systemic and mucosal immune responses in mice and for building a two-layered defense system against *Shigella* infection. Moreover, our study also reveals strong Th1 type protective immune response in terms of IFN-γ and TNF-α release. So, both humoral and cell mediated immunity may be playing crucial role in the OmpA induced protective immune responses.

In conclusion, we demonstrate that the expressed OmpA of *S. flexneri* 2a induces strong immunogenicity and protective efficacy in a murine model of intranasal challenge and represents a promising and effective candidate for use in the future development of a subunit vaccine against shigellosis in human beings.
